# Development and Evaluation of the CASTLE Trial Online Sleep Intervention for Parents of Children with Epilepsy

**DOI:** 10.3389/fpsyg.2021.679804

**Published:** 2021-07-02

**Authors:** Luci Wiggs, Georgia Cook, Harriet Hiscock, Deb K. Pal, Paul Gringras

**Affiliations:** ^1^Centre for Psychological Research, Department of Psychology, Health and Professional Development, Faculty of Health and Life Sciences, Oxford Brookes University, Oxford, United Kingdom; ^2^Health Services Research Unit, Royal Children's Hospital, Melbourne, VIC, Australia; ^3^Centre for Community Child Health, Murdoch Children's Research Institute, Melbourne, VIC, Australia; ^4^Department of Paediatrics, The University of Melbourne, Melbourne, VIC, Australia; ^5^Department of Paediatric Neuroscience, Evelina London Children's Hospital, London, United Kingdom; ^6^Department of Basic and Clinical Neuroscience, Institute of Psychiatry, Psychology and Neuroscience, King's College London, London, United Kingdom; ^7^Medical Research Council Centre for Neurodevelopmental Disorders, King's College London, London, United Kingdom; ^8^Department of Paediatric Neuroscience, King's College Hospital, London, United Kingdom; ^9^Children's Sleep Medicine, Evelina London Children's Hospital, London, United Kingdom; ^10^Women and Children's Institute, King's College London, London, United Kingdom

**Keywords:** intervention, epilepsy, online, sleep, e-health, telehealth, children, parents

## Abstract

**Introduction:** Many of the sleep problems experienced by children with epilepsy (CWE) have the same behavioural basis as common sleep problems seen in typically developing (TD) children. Behavioural sleep interventions (BSIs) are widely used to treat these sleep problems in TD children and are hypothesised to be effective for CWE. However, specific considerations need to be addressed and incorporated into a BSI for CWE to ensure the intervention is tailored to this population's needs. This paper details developing and tailoring an online BSI for parents of CWE, to be used in the CASTLE (Changing Agendas on Sleep, Treatment and Learning in Epilepsy) Sleep-E clinical trial.

**Method:** In phase one, two existing theory-driven paediatric BSIs were adapted into a novel online behavioural sleep intervention (CASTLE Online Sleep Intervention or COSI) which specifically incorporated the needs and requirements reported by nine parents of CWE. Scoping their needs included conducting interviews with three CWE so that they could contribute to the overall intervention content. In phase two, six of these parents evaluated COSI, reviewing and feeding back on COSI until parental approval for content and functionality was achieved.

**Results:** In phase one, a range of adaptations was made to the content and presentation of standardised intervention material to acknowledge and emphasise the key seizure-specific issues to ensure COSI best met parents of CWE's needs. Adaptations included embedding parent and child experiences in the intervention, including particular information requested by parents, such as the links between sleep and seizures and managing child and parental anxieties around sleep, as well as developing functionality to personalise the delivery of content. In phase two, parents confirmed that they found the final version of COSI to be functional and appropriate (after one round of review) for use by parents of CWE and that 100% would recommend it to other families who have CWE.

**Discussion:** It is hoped that the use of evidence-based BSIs, adapted to consider salient epilepsy-specific factors, will increase parent-engagement, COSI's relevance for this particular patient group and overall efficacy in improving sleep in CWE. The effectiveness of COSI will be tested in the CASTLE Sleep-E clinical trial (https://castlestudy.org.uk/).

## Introduction

Epilepsy is a common neurological condition, characterised by a tendency for recurrent seizures, and is primarily a childhood-onset condition (Hauser and Hesdorffer, 1990). Epilepsy has a population prevalence of 1%, with ~63,400 children and young people under 18 years old affected in the UK (Joint Epilepsy Council, 2011). Seizures have been found to account for 5% of the total of childhood emergency admissions (Armon et al., 2001) and epilepsy in children is commonly comorbid with cognitive, learning and behavioural difficulties which can lead to academic underachievement (Children with Epilepsy in Sussex Schools, 2014).

Sleep disturbance is a surprisingly common comorbidity for children with epilepsy (CWE) with sleep problems occurring more often than in typically developing (TD) children (Owens and Mindell, 2011). Even in the absence of nocturnal seizures, sleep problems as reported by parents are 12 times more common in 4–10 year old CWE than in children without epilepsy (Gutter et al., 2013).

There are a range of possible negative implications of the sleep problems experienced by CWE. These include daytime sleepiness, impaired cognitive functioning, behaviour, and quality of life (Stores et al., 1998; Maganti et al., 2006; Owens and Mindell, 2011). There are also a range of negative implications for the sleep of parents of CWE (Larson et al., 2012), with mothers of CWE being seven times more likely to suffer from sleep disturbance than parents of TD children (Shaki et al., 2011). Further, evidence suggests parents of CWE's sleep duration is impacted, on average obtaining only 4 h of overnight sleep with fragmented parental sleep also positively related to poorer maternal well-being and marital satisfaction (Cottrell and Khan, 2005).

Sleep and epilepsy have been described as “unfortunate bedfellows” due to their complex bi-directional relationship (Gibbon et al., 2019). This is because sleep may be disrupted due to seizures experienced during sleep, comorbid sleep disorders and/or effects of antiseizure medications. Sleep disturbance can, in turn, lower seizure threshold (da Silva Sousa et al., 2005; Gibbon et al., 2019). Many types of epilepsy have specific associations with sleep, including specific stages of sleep and circadian pattern (Loddenkemper et al., 2011; St Louis, 2011). In rolandic epilepsy (RE), the most common type of epilepsy in childhood (Cavazzuti, 1980; Sidenvall et al., 1993), seizures are most often nocturnal (Jain and Kothare, 2015), commonly occurring just after falling asleep or shortly before waking. The adverse associations with poor sleep in CWE mean that sleep issues should be an intervention target which, if successfully addressed, may have positive benefits for various child and parental outcomes, including reduced seizures.

Many of the same sleep problems experienced by CWE are commonly seen in TD children, including problems with sleep initiation (settling and falling to sleep) and/or maintenance (night or early morning waking). These disorders (as distinct from more physical sleep disorders like sleep apnoea) are commonly amenable to behavioural sleep interventions (BSIs). The aim of these interventions is to provide parents with practical strategies to modify their practises around their child's sleep so that they can support their child to “learn” appropriate sleep behaviours and, if necessary, to “unlearn” inappropriate sleep behaviours. They typically also include parental education about normal sleep and developmental aspects of sleep, as well as provide advice about good sleep hygiene, which refers to practises that promote good sleep such as regular bedtimes and limiting use of LCD screens in the bedroom (Meltzer and Mindell, 2014). BSIs have been found to be effective in randomised controlled trials for young (under 5 years) TD children, and in autism and attention-deficit hyperactivity disorder (ADHD) populations, including up to age 12 years (Mindell et al., 2006; Johnson et al., 2013; Hiscock et al., 2015). Because of this body of evidence it has been proposed that BSIs could also be modified effectively for children with epilepsy (Gibbon et al., 2019) although, to the authors' knowledge, there have been no trials which have specifically evaluated this. BSIs can be delivered in a variety of modes including face-to-face (Hiscock et al., 2015), by telephone (Stuttard et al., 2015), by written information (Gringras et al., 2012), and online/app based (Mindell et al., 2006; Espie et al., 2012).

Sleep problems in children with epilepsy are often not targeted or evaluated in clinical care or trials. For example, current NHS services for CWE are focussed on addressing seizures and are often under resourced to allow adequate time to support a BSI. The Changing Agendas on Sleep, Treatment and Learning in childhood Epilepsy (CASTLE) group consisted of lay members and professionals who contributed to the design and planning of a broad programme exploring sleep, treatment and learning in epilepsy, culminating in a national trial for an online BSI for parents of children with RE (CASTLE Sleep-E clinical trial. See: https://castlestudy.org.uk/). An online BSI was chosen for evaluation in this trial due to the RCT evidence of the efficacy of such an approach for similar childhood (Mindell et al., 2006) and adult (Espie et al., 2012) sleep problems in diverse populations. In addition, parents of CWE frequently use the internet to try to access information and support, and others have noted the need for the development of high-quality online resources for parents (Jones et al., 2019). Importantly, an online BSI (i) offers cost effectiveness, (ii) addresses many logistical considerations of rolling out and delivering nationwide interventions, (iii) aligns with future NHS trends in telemedicine, minimising NHS staff burden and the need for staff training to deliver the intervention, and (iv) has the potential to ultimately maximise patient access to the intervention. A further advantage of the online approach is that it can also generate objective process measures of frequency and pattern of access to the materials, and, for example, scores on quizzes (as a proxy for understanding of the materials) to help inform scientific understanding about causal pathways behind a complex intervention.

Despite the good quality evidence supporting the use of BSIs, and online delivery, for children's sleep problems there are special seizure-specific considerations that could usefully be acknowledged to help ensure that the online BSI best meets the needs of this particular parent group (Cook et al., 2021). There are well-described benefits of co-creating such resources with carers and children who are “experts by experience” (D'Alessandro and Dosa, 2001; Carman et al., 2013). Our study represents an attempt at this best practise and our methods describe this process of development (phase one) and initial parent evaluation of content and functionality (phase two) of the CASTLE Online Sleep Intervention (COSI), for subsequent use in the Sleep-E clinical trial.

## Method

### Design

Phase one describes the development of COSI, an online BSI for parents of CWE, which incorporates (i) information about evidence-based BSI strategies and (ii) adaptations and additions suggested by parents of CWE. Specific requirements for this group were identified via interviews with parents of CWE (Cook et al., 2021). After an initial draft of COSI was developed by the researchers, phase two could begin. In this, parents evaluated the content and functionality of this draft during a 2-week period and then reported on the acceptability of content and functionality of the intervention website. This iterative drafting and evaluation cycle was planned to continue until parental approval (defined below under Measures) was achieved. It should be noted that in evaluating COSI parents were not asked to implement any BSI, but rather to visit the intervention website, read the text, watch the videos, see if they could easily navigate around the website etc. The effectiveness of COSI as a BSI will be assessed in the forthcoming Sleep-E clinical trial.

### Participants and Recruitment

#### COSI Development (Phase One)

Epilepsy specific COSI adaptations were informed from interviews with nine mothers of CWE (six males, age range 5–15 years, median=10 and mean=10.3, SD=2.9), who all lived with their CWE. Further details of their children's sleep and epilepsy are available in the Supplementary Appendix 1 but, in summary, of the children, five had Benign Rolandic Epilepsy (1 atypical), 2 had focal seizures, 1 generalised seizures and 1 unspecified. Two had been diagnosed with epilepsy < 1 year ago, 2 children between 1 and 3 years ago and 5 children >3 years ago. All the children in this sample had experienced a range of sleep problems, both currently and in the past. Participants were recruited for telephone or video interviews, between March and July 2018, via online advertisements placed on the websites of epilepsy organisations and charities and the CASTLE study and researchers' university websites.

In addition, three children with RE (2 males aged 10–14, 1 female aged 10), recruited via the CASTLE study patient and public involvement and engagement group, participated in the development of COSI in a specific way; interviews about their sleep experiences (conducted in November 2018) were used to contribute to the content of the online BSI. Anonymised quotations from the children featured in video animations created for and embedded into COSI (see Procedure, COSI Development, below).

#### Evaluation Study (Phase Two)

Six of the nine mothers of CWE (four males, ages ranging 5–15 years, with median=10, mean=10, SD=3.69) who had participated in the interviews also participated in the evaluation element of the study where they reviewed the developed COSI and provided their feedback about the acceptability of content and functionality (between 13th and 28th March 2019).

### Measures

A COSI Evaluation Questionnaire was developed by the research team and hosted via Qualtrics (Qualtrics, Provo, UT) (see Supplementary Appendix 2). This allowed for parental quantitative assessments of the content and functionality of the COSI website in the evaluation study (phase two). There were four elements to this questionnaire: (i) the functionality of COSI, (ii) the acceptability of the content, (iii) the acceptability in terms of length and content of the embedded COSI parent feedback scale (which will be used to assess parents' experiences of COSI when it is used in the CASTLE Sleep-E trial) and (iv) allowing parents opportunity to make any further comments.

#### The Functionality of COSI

Parents were asked to rate (on a 5 point Likert scale of “Always,” “Often,” “Sometimes,” “Rarely,” and “Never”) the frequency with which they could log on, access the webpages' content, see and hear the videos, access and respond to the quizzes, print off any materials they wanted and navigate easily around the intervention website. If parents responded with anything other than “Always” they were asked to explain any problems in an open text response box. Using the same 5-point scale parents were also asked if they would recommend COSI to other families of CWE.

#### The Acceptability of Content

For each of the possible suggested 20 behaviour change techniques (see Supplementary Appendix 2, section 2) which were included in COSI, parents were asked to report how acceptable they thought it was to include the technique as a suggestion (even if they personally might not use the technique). As well as explicitly listing and describing each of the techniques which the researchers considered to be included in COSI an “Other” option was also included in case there was further content which parents perceived to be a suggested technique. Parents rated these on a 5-point Likert scale of “Very acceptable,” “Acceptable,” “Neither acceptable nor unacceptable,” “Unacceptable,” and “Very unacceptable.” If they responded “Unacceptable” or “Very unacceptable” they were asked to explain their reasons in an open text response box.

#### The Acceptability of the COSI Parent Feedback Scale

Parents were also asked to rate the acceptability of the embedded COSI feedback scale in terms of the burden of the time taken to complete (on a 5-point Likert scale of “Far too long,” “A little too long,” “About right,” “A little too short,” and “Far too short”) and the understandability of the language and phrasing used (“Very difficult,” “Difficult,” “About right,” “Easy,” and “Very easy,” with open text response boxes to expand as required) as well as whether there were other questions which they felt should be included (“Yes” or “No,” again with open text response boxes to expand as appropriate).

#### Additional Comments

Finally, there was an open text box where parents could add any additional comments about COSI.

Based on parents' responses to this questionnaire, our definition of “parent approval” was as follows:

(i) All parents reported they were able to access all elements of COSI as intended.
(ii) >65% positive rating (“Always” or “Often”) on a “Friends and Family” style 5 point Likert measure answering: “How likely are you to recommend this sleep programme to other families who have children with epilepsy?”
(iii) >65% parents report acceptable burden (“About right”) of embedded COSI parent feedback scale.

### Procedure

Ethical approval was obtained through the Oxford Brookes University's Research Ethics Committee (UREC approval 171108).

#### COSI Development (Phase One)

The key content (informational and instructional content) of two existing BSIs used in randomised control trials (RCTs) of children with ADHD (Hiscock et al., 2015) and Autism (Johnson et al., 2013) was identified from the published reports and clarification with the authors as required (see Table 1, columns 1–3 for a summary of each BSI) and this core content was drafted into text format. The BSI content from these studies was selected for a number of reasons, including: (i) these previous studies are evidence-based level 1 studies with good effect sizes; (ii) they utilise well-defined interventions that are easy to tailor to specific family needs and goals; (iii) their relative brevity; (iv) their established effectiveness in children with neurodevelopmental disorders; and (v) their suitability to be adapted to an online intervention. Both existing BSIs comprise a number of reliable combinations of behavioural change techniques (BCT) defined as “systematic procedures included as an active component of an intervention designed to change behaviour” (Michie et al., 2011).

**Table 1:** Overlap and extension of informational and instructional content from existing BSIs and in COSI.

	**Johnson et al., 2013**	**Hiscock et al., 2015**	**COSI**
Normal sleep information		✓	✓
Sleep and condition-specific information			✓
Common sleep problems information		✓	✓
Explanation of basic behavioural principles	✓		✓
Prevention techniques (sleep hygiene recommendations, schedules and routines)	✓	✓	✓
Behavioural sleep management techniques for bedtime resistance, delayed sleep onset, sleep onset association, night waking and early waking (details below)	✓	✓	✓
Extinction	✓	✓	✓
Graduated extinction	✓	✓	✓
Bedtime fading	✓	✓	✓
Scheduled wakings	✓		
Stimulus control	✓	✓	✓
Reinforcement	✓	✓	✓
Behavioural techniques to address anxiety related insomnia or night fears (details below)	Optional	✓	✓
Visual imagery		✓	✓
Relaxation		✓	✓
Teaching “brave” skills	✓		✓
Systematic exposure	✓		✓
How parents can reassure	✓		✓
Additional advice for managing sleep in children with co-occurring conditions	✓		✓
Strategies for maintenance of behaviour change	✓		✓
Behaviour compliance training	Optional		
Dealing with sleep walking, sleep terrors, and nightmares		✓	✓
Troubleshooting advice	✓	✓	✓
Information to help address parental anxieties and concerns			✓
Other forms of content (details below)			
Printed handouts	✓	✓	✓
Videos modelling use of strategies	✓		
Role play	✓		
Homework activities	✓		
Videos sharing experiences of parents and children			✓
Quizzes to test knowledge			✓
Direction to additional relevant online resources			✓

Based on the results of a previous interview study with parents of CWE (Cook et al., 2021) parent views about factors which should be integrated into, and issues that would need to be acknowledged as part of, any online BSI were described (see Table 2, column 1 for a summary). BSI content, delivery and usability were then adapted to address this parental feedback, to produce a resource which used evidence-based strategies and acknowledged the topics identified through patient and parent involvement. It was hoped this would result in a resource that parents found helpful and acceptable, and that with the contribution of CWE and their parents to its development made clear, would encourage parental trust and engagement with COSI.

**Table 2:** Illustration of how different themes reported by parents were integrated into COSI.

**Theme**	**How integrated into COSI**
Other parents' views and experiences	•Anonymised quotations from interviews with parents were used in animated videos embedded throughout COSI to weave in other parents' views and experiences (see Figure 3 for an illustration). This represented the “personal” experiences of parents of CWE and contributed to providing support, and hopefully add credibility to, the material and ideas presented as part of COSI content •The inclusion of a frequently asked questions (FAQs) page which addresses key topics raised by parents such as what to do if child is unwell, child sleeps in multiple households, and approaches for how to manage parents' own worries (module K)
Change over time	•Parents were clearly advised that they should select options most relevant to them at the specific time of review and that this may change over time •Sleep management techniques were presented carefully to ensure factors that might impact their choice of approach were identified and acknowledged •The presentation of a range of evidence-based intervention techniques (modules G, H, I, and J) allowing parents to choose which option may be most suitable to them at different time points
Range of management options	•In any modules where behaviour-change techniques were presented (module G, H, I, and J) a variety of different evidence-based techniques were provided •In any modules where behaviour-change techniques were presented these were worded carefully to present the family with options rather than “instructions”
Personalisation of information	•Functionality embedded partly through a “sleep screener” questionnaire (module C) where recommended modules were highlighted based on parents' responses to the sleep screener questionnaire to help personalise recommended modules for parents to review •Parents completed this screener based on their child's sleep behaviour allowing them to feel their individual child's sleep was being considered •Encouraging parents to select from the range of behaviour-change approaches presented (module G, H, and I predominantly) also encouraged parents to personalise their own use of COSI •COSI provided specific evidence-based information and strategies for parents whose child may have comorbid conditions (module F)
Child anxiety around sleep	•Inclusion of a module (I) specifically aimed at supporting parents to manage sleep-related anxiety and night-time fears in their child •Also included was information (module J) designed to help parents address specific night-time behaviours (sleep walking, sleep terrors and nightmares) with their child
Practical sleep intervention suggestions	•The BSIs upon which COSI was based include a range of practical strategies that parents can select from and apply to support their child to “learn” appropriate sleep behaviours and, if necessary, to “unlearn” inappropriate sleep behaviours. •Presented in a clear step-by-step fashion making them easy to follow and implement •Inclusion of a troubleshooting section (module K) to help parents practically deal with common issues around implementing BSIs and also practical advice about how to maintain any sleep improvements
General sleep information	•The development of COSI itself was designed to address parents' desire for knowledge and information around sleep •Particular areas about which parents desired information were explicitly incorporated into COSI (i.e., general background information about sleep (module A), the relationship between sleep and seizures (module B), sleep hygiene tips (module D), understanding common sleep problems and possible approaches to managing or improving CWE's sleep (module E, G, H, I, and J)
Parental anxieties and concerns	•COSI content clearly acknowledged areas of parent anxiety (e.g., about sleep, epilepsy and intervention approaches) throughout •COSI specifically provided some information about parental worries and sleep in the FAQs page (module K) •COSI identified further useful resources of help and support for parents around possible areas of anxieties and concerns (module L)
Help, support and reassurance around sleep	•The purpose of developing COSI was to provide help, support and reassurance to parents around child sleep •Careful consideration was given to the wording throughout COSI to ensure it was written in a non-judgmental and supportive manner •Including other parents' experiences (as this was reported to be thought helpful, supportive and reassuring for them) •Acknowledging the possible challenges of intervention approaches, helping parents feel informed and supported in making the most appropriate decision for their individual child/family •Providing general sleep information to reassure parents about variability of child sleep •That there are techniques designed to improve sleep, if required, would be supportive and reassuring for some parents
Include child in intervention	•Anonymised quotations from interviews with CWE were integrated throughout COSI in animated videos, reflecting their perspectives (see Figure 4 for an illustration)

To integrate the BSI content and parents' views the researchers reviewed the themes identified from the parent interviews and, for each, considered ways in which this could be included in COSI. This resulted in adapting some of the draft text content and adding new text content (see Table 1, column 4 for a summary of COSI content, in relation to that of the content of the existing BSIs from which it was developed), including producing video animations to embed into COSI, featuring quotes from parents. In response to parents' suggestion that children's perspectives should be included in COSI three CWE were interviewed and asked how they felt about sleep, about any problems they had with sleep (generally and related to their epilepsy) and to tell us about things they had found helpful, or not, for their sleep (see Supplementary Appendix 3). Quotes from the children, illustrating their opinions and experiences, were also included in the video animations.

Where appropriate, text content was reviewed for acceptability by paediatric epilepsy specialists. For example, the topic of parent concerns about Sudden Unexplained Death in Epilepsy (SUDEP) exemplified the need to combine research [i.e., evidence-based guidelines about SUDEP based on Harden et al. (2017)], clinical (comments on our draft text about SUDEP from three paediatric epilepsy specialists) and patient and parent involvement and engagement to recognise and respond to parents' concerns so that they felt willing and able to engage with any suggested strategies for sleep intervention.

Parent suggestions related to the presentation and usability of the website were addressed (see Table 2 for details of how parents' suggestions were integrated into COSI) and the functionality and presentation of the intervention website was reviewed and tested by the researchers.

#### Evaluation Study (Phase Two)

Following this development, access to the COSI intervention website was provided to parents who had participated in the interview study. Participants were emailed a password protected weblink to access COSI and were asked to review and interact with the website, exploring the content and functionality. Participants were then asked to provide quantitative feedback via the COSI Evaluation Questionnaire, access to which was also sent as a weblink. Parents were offered an opportunity for further discussion with researchers to provide additional feedback however, no parents requested follow-up discussion. This process of parent evaluation and re-drafting was to be continued until parent approval (as defined above) was obtained. However, parental approval was achieved after the first parent evaluation.

## Results

### COSI Development (Phase One)

As shown in Table 1, core information and a range of standardised evidence-based techniques to address sleep problems in children, as used in the existing BSIs, were included in COSI. COSI was extended to include additional content highlighted as pertinent to the parents of CWE in the previous qualitative study exploring what parents of CWE wanted from an online BSI for this clinical group (Cook et al., 2021). The main content additions included (i) embedding parent and child experiences in the intervention, (ii) including information about sleep and seizures and acknowledging epilepsy-specific considerations, (iii) including information about nightmares, sleep walking and sleep terrors and (iv) including information about managing child and parental anxieties around sleep. Further, delivery and presentation of material was tailored to meet parents' identified needs, within the constraints of the online delivery platform; for example “personalising the presentation of the material” was achieved by recommending pertinent sections of COSI in response to parents' answers to a screening questionnaire where they reported on their child's sleep and offering parents a “range of management options,” was supported by careful choice of words when describing the techniques to parents, so that they felt they were being offered suggestions rather than instructions. See Table 2 for details how the key themes reported by parents were specifically acknowledged, addressed and or integrated in COSI.

Some aspects of the existing BSIs were not incorporated into COSI due to either not being possible for the current online mode of delivery (e.g., role play, homework), not identified as a priority by the parents (e.g., videos modelling the use of strategies) or possibly unhelpful for this group (i.e., suggested use of scheduled wakings, which could increase sleep disturbance in the short term). The addition of quizzes and links to other online resources were included, not because they had been spontaneously suggested by parents but because they were obvious possible additions afforded by the online platform and, when questioned about their possible inclusion, parents reported they could be welcome additions and gave useful advice about their functionality (e.g., quizzes should be optional). COSI was a bespoke web application, purpose built for CASTLE. It was designed to allow for easy presentation of modular content, embedded animations, quizzes and data capture. As a web-based tool optimised for different devices across all platforms it was also easily accessible to all families.

**Figure 1:**
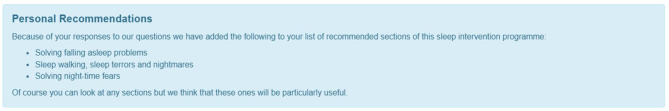
Personalised recommendations for modules based on parental responses to screening questionnaire.

COSI included 11 modules as shown in Table 3. The first 3 COSI modules (A, B, and C) were compulsory as they provided important information relevant to subsequent sections. Parents were required to complete each of these sequentially and were unable to access later modules until each was completed. Having completed module C (screening questionnaire) all subsequent modules were made available to parents and could be accessed at any time although, as explained, to personalise the intervention, a list of “recommended modules” specific to their child's sleep was derived based on their responses to the screening questionnaire (see Figure 1).

**Table 3:** COSI module names, descriptions and compulsory or recommended status.

**Module**	**Module name**	**Outline content**	**Compulsory or recommended**
A	What is sleep and why is it important	Education about normal sleep physiology and processes	Compulsory
B	Sleep and seizures: a vicious cycle	Information about the relationship between sleep and seizures	Compulsory
C	Personalising this advice for your child	A sleep screening questionnaire to identify key areas of concern or problems around individual child sleep	Compulsory
D	Tips on sleep hygiene for everyone	General advice about key aspects of sleep hygiene	Recommended for all
E	Advanced sleep behaviour training	Introduction to principles of behavioural sleep interventions	Recommended for all
F	Learning difficulties, ADHD and Autism spectrum disorders	Advice for parents of children with comorbid conditions	Recommended to parents who highlighted (in module C) their child may have comorbid conditions
G	Solving falling asleep problems	Sleep intervention options for typical falling asleep problems	Recommended to parents who highlighted (in module C) their child may have problems falling asleep
H	Solving difficult night wakings and early morning waking	Behavioural techniques to address typical night or early waking problems	Recommended to parents who highlight (in module C) their child may have problems with night or early morning wakings
I	Solving night time fears	Behavioural techniques to address typical night time fears	Recommended to parents who highlight (in module C) their child may have problems with night time fears
J	Sleep walking, sleep terrors, and nightmares	Information about these specific sleep disorders, what causes them and how to identify and manage different conditions	Recommended to parents who highlight (in module C) their child may have problems with sleep walking, sleep terrors, and/or nightmares
K	Troubleshooting and maintaining good sleep	How to deal with common issues, such as the child being ill or parents' disagreeing about how to manage sleep and also advice about how to maintain any benefits	Recommended to all
L	Resources	Links to additional resources of support, information and advice relating to sleep	Recommended to all
M	COSI parent feedback scale	Questionnaire in which parents are asked to report on their experiences of using COSI	Recommended to be completed by all

**Figure 2:**
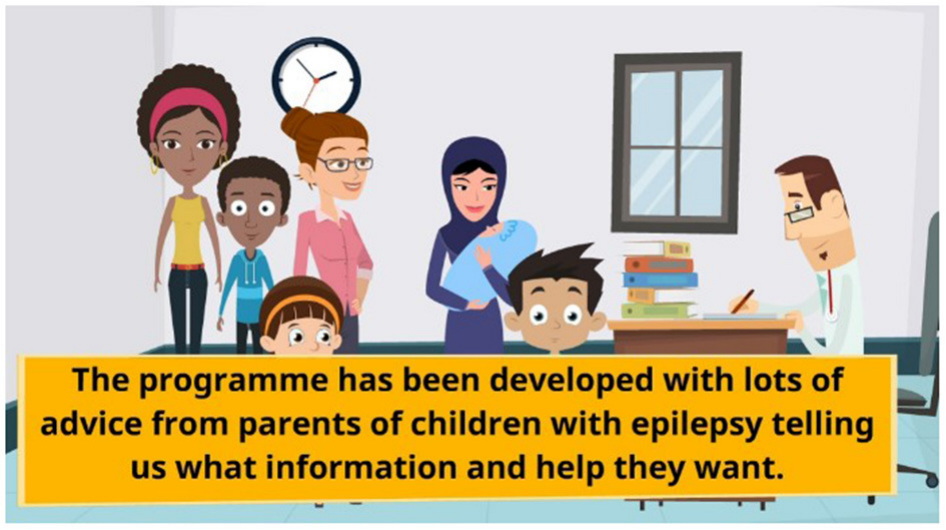
A still of the animations used in COSI. Illustration is from the introduction to the online BSI.

Modules D, E, K, and L were recommended to all parents alongside any of modules F, G, H, I, J, as appropriate. The COSI parent feedback scale (module M), to be completed at the end of parents' use of COSI, was embedded into the intervention website; this would allow for exploration of parents' views about the use of the COSI and the suggested intervention techniques. The structure of each information module (A, B, D-J) was the same, starting with a general introduction to the topic which was both written and also presented in an animated video (see Figure 2 for a still of animation content).

**Figure 3:**
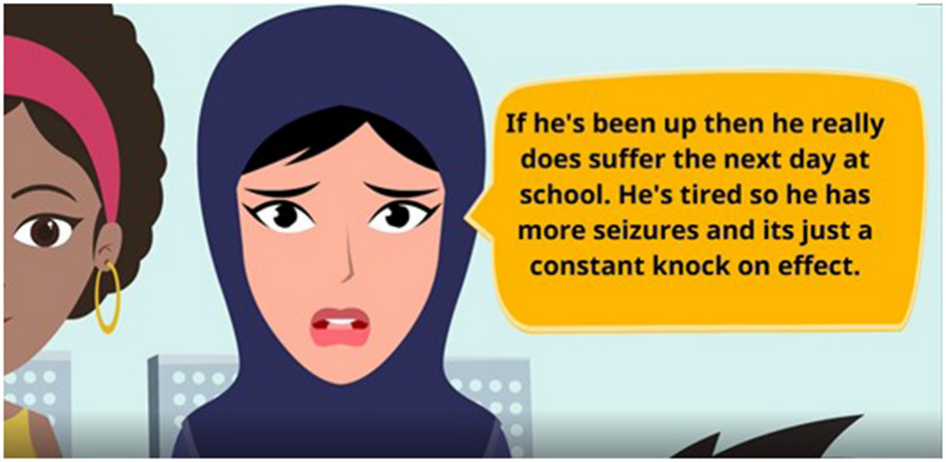
Still from COSI animation demonstrating integration of parental perspective (quote).

**Figure 4:**
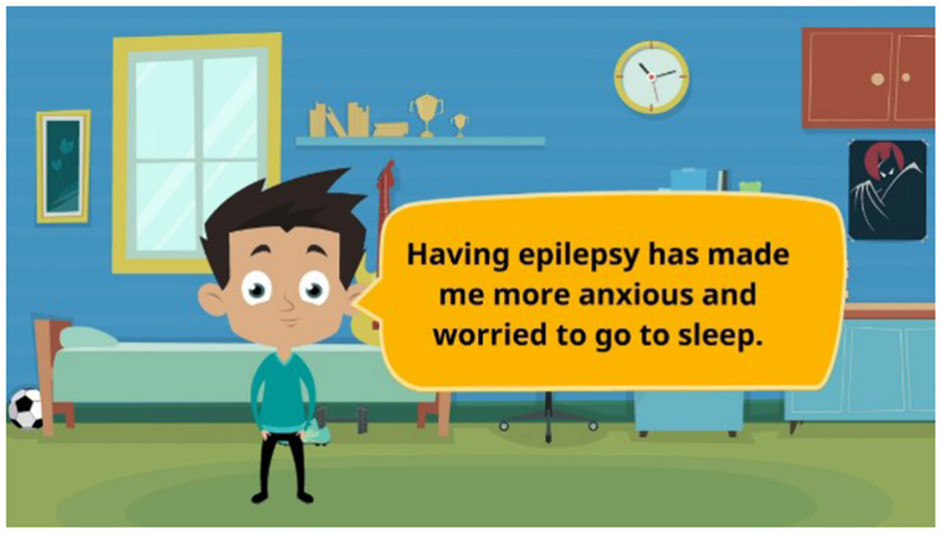
Still from COSI animation demonstrating integration of child perspective (quote).

The “scripts” for the animations included quotes from parents and CWE (spoken by actors) so that, as requested by parents, the experiences of the families, and not just clinicians' views, were embedded into COSI (see Figures 3, 4 for stills from animations illustrating parental and child quotes). Following the introduction for each module, a series of subheadings could be clicked to expand and reveal information and images. Common parent concerns related to the module topic were also included as subheadings. Throughout COSI hyperlinks to other relevant modules, or to other external websites, resources and videos were provided where appropriate. At the end of each module there was a quiz. If a parent did not answer correctly, the correct answer was shown.

Some modules gave general educational information, for example about normal sleep (module A) or, tailored to this particular clinical group, the links between sleep and seizures (module B). Module C included the screening questionnaire, enabling the COSI experience to be personalised by directing parents to modules which were particularly relevant for them, something which parents had reported was highly desirable. If appropriate they were guided to speak to their clinician about any sleep issues which were screened for but not addressed in COSI e.g., symptoms suggestive of a sleep related breathing disorder.

**Figure 5:**
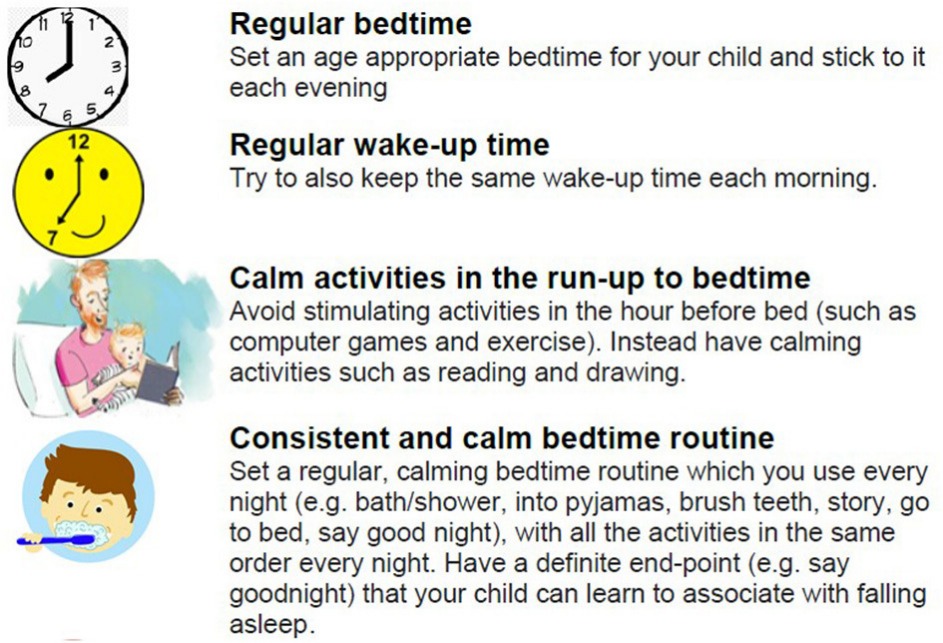
Image of the type of advice and presentation of material for module D, sleep hygiene tips.

Some modules provided general advice and guidance relevant for all. For example, module D provided details about basic sleep hygiene practises and module E gave details about the underlying principles of behaviour change. See Figure 5 for an example of the content of module D. Some modules provided targeted advice for specific scenarios. For example, module F provides advice and guidance about sleep and sleep interventions for parents whose children had other co-occurring conditions, such as ADHD and Autism spectrum disorders. The inclusion of material around co-occurring conditions was considered appropriate for this target group, to help parents feel supported and to encourage them to feel that the intervention was relevant and personal for them and their child.

**Figure 6:**
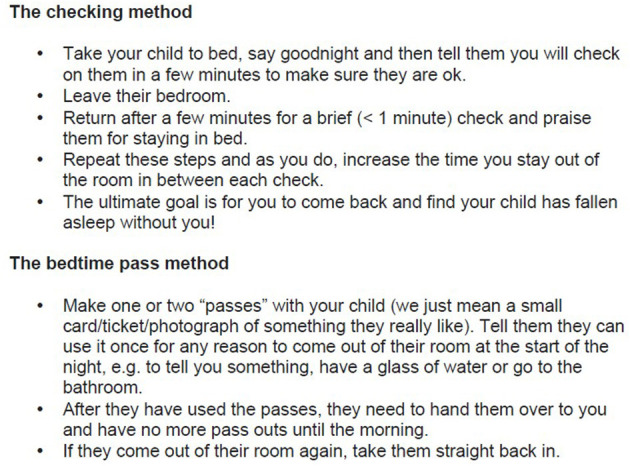
Example content of module G, solving falling asleep problems. Multiple evidence-based strategies presented.

For the modules giving information about behaviour change techniques (modules G, H, I, and J), all addressed a different “type” of sleep problem: falling asleep problems; difficult night or early morning waking; night-time fears; sleep walking, sleep terrors and nightmares. Each behaviour change module presented a range of possible strategies, to meet parents' desire for a variety of options from which they could choose which approach to use. As mentioned, all behavioural change methods in COSI were carefully worded as suggestions for the family to consider and were presented in a clear step-by-step fashion making them practical to follow, which was an additional key requirement reported by parents. For example, see Figure 6 for some of the behaviour change techniques presented to parents if they have a problem with their child stalling at bedtime (also known as limit setting disorder). The explicit inclusion of material relating to addressing children's night-time fears (module I) and material relating to sleepwalking, sleep terrors and nightmares (module J) were included as parents explicitly requested help with managing or addressing their child's anxiety around sleep and requested support with these common child sleep problems. It was made clear that some of the strategies might be easy to start putting into practise at any time but for others they would need to judge when it felt right for them and their child to acknowledge the issue of changes over time, to emphasise how the material could be “personalised” and also to encourage informed parental choice.

**Figure 7:**
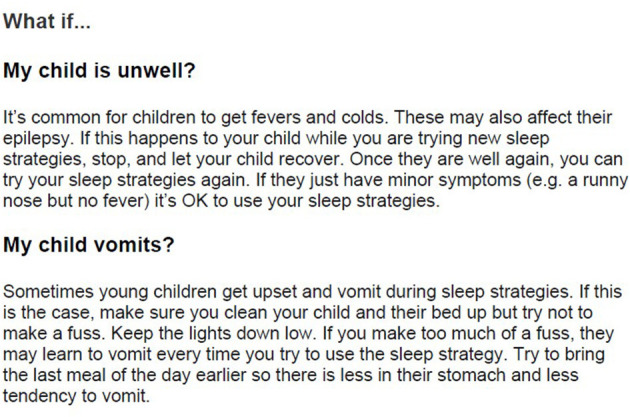
Example of COSI content from module K.

A benefit of the online delivery was that a range of relevant links could be provided as resources for parents (module L) to help them feel further supported. These included information relevant to child sleep, epilepsy and also support for parents themselves, about coping when their own worries affected their sleep. In module K, common concerns related to implementing intervention (e.g., when the child is ill or parents disagree about management strategies) were raised alongside suggestions for how to maintain any gains over the longer-term (see Figure 7). It was hoped this inclusion would help to meet parents' desire for both practicality and support in dealing with any issues that arose.

Also included in this module (K) was an attempt to address one component of a theme which featured prominently in the parent interviews; that parents commonly come upon ideas for things that might help their child's sleep from anecdotal reports from other parents on social media and internet forums and that they valued this resource. Whilst we did not feel that we could endorse potential management tools and strategies for which there was no strong evidence base, we did think it was important not to ignore that parents used and valued these suggestions. In response, we decided to give parents some information to empower them about how to approach and make decisions about whether they should use unproven interventions.

The end of COSI included a parent feedback scale (module M) which will be used to evaluate COSI in the Sleep-E trial. This will ask parents to report on how easy the online intervention was to use (i.e., ease of logging on, accessing written, video and quiz content and ability to print off any required materials). Parents will also be asked to indicate which techniques they used and, if they report using specific techniques, how useful they found them to be. Alternatively, if parents report that specific techniques were not put into practise, they were asked to report the reasons for this. Finally, parents will be asked to read and rate their agreement with statements reflecting the thoughts and feelings parents may have when faced with a child that is not sleeping. These items were derived from an age-appropriate version of the Maternal Cognitions about Infant Sleep Questionnaire (Morrell, 1999), modified by excluding the three items asking about night-feeding (as used in Montgomery and Wiggs, 2015).

COSI was developed to collect quantitative, descriptive, e-learning data about parents' use of the intervention website in the Sleep-E trial. This will assess how many modules parents have looked at; how many times they have viewed a module; the duration of time on the website and individual modules, as well as parents' scores on quizzes and questionnaires. This allows for a “dose-related” exploration of the impact of parental engagement and understanding on overall efficacy.

The time required for parents to read and engage with each module will obviously vary depending on a number of factors (including whether parents chose to engage with hyperlinks to external resources and how many times they answer the quiz questions) but, as a descriptive indication of the demands COSI placed on parents, the text for each module could be read in 5–10 min at most and each animated video lasted for 1–2 min.

### Evaluation Study (Phase Two)

After reviewing COSI, parent feedback assessed by the COSI Evaluation Questionnaire (*n* = 6) was unanimously positive. All parents reported they were able to access and use the online intervention as intended. One parent reported a minor error in a video animation (sub-optimal timing of text and audio presentation), which was corrected. All parents approved of COSI and reported they would recommend this sleep programme to other families who have children with epilepsy. All techniques were considered to be “acceptable” or “very acceptable” suggestions. All parents reported the embedded COSI parent feedback scale to be of acceptable burden (all “about right”) and understandable (all “easy” or “about right”). Finally, whilst not a parent-approval metric, the researchers noted the “back-end” of the system successfully captured the desired e-learning analytics for all participants (e.g., time logging on, pages visited, quiz answers recorded etc.).

Following phases one and two, and with the criteria for “parent approval” exceeded, COSI was subsequently approved by the CASTLE trial programme board for use in the NIHR-funded CASTLE programme where its effectiveness will be evaluated in the Sleep-E trial (https://castlestudy.org.uk/).

## Discussion

We have described the co-creation of COSI, an online BSI for parents of CWE, adapted from the informational and instructional content of existing evidence-based BSIs. In doing so we took account of the special considerations, of content and delivery, for CWE and their families to help ensure COSI best met their needs. Parents' reviews of the content and functionality of COSI were universally positive and the parental “approval metric” criteria was exceeded. Of course, the clinical effectiveness of COSI cannot be determined from the current results.

Previous research has highlighted the need for high-quality online resources for parents of CWE due to the widespread reported use of the internet to access information and support (Jones et al., 2019). Whilst there are many general online sources of information about sleep available for parents, the large quantity of information can feel overwhelming for parents (Walsh et al., 2015; Cook et al., 2021) and decisions about whether or not to engage with resources can be even more difficult for families where children have particular needs or medical conditions as the advice offered may not obviously appear to be relevant or appropriate for their family circumstances (Zeng and Cheatham, 2017). COSI was designed to address this problem, in relation to the sleep of CWE, by providing salient information, evidence-based management suggestions and delivery to families in a way that accorded with parents' needs and preferences, as identified from interviews with parents of CWE.

The range of sleep-related behaviour change techniques included were not unique to COSI, but rather were specifically chosen because they had been previously demonstrated to be effective when used in various paediatric populations, including children with neurodevelopmental disorders (Mindell et al., 2006; Johnson et al., 2013; Hiscock et al., 2015) and when delivered in various ways, including online (Mindell et al., 2006; Espie et al., 2012). However, the evaluation of their use specifically in CWE is original and will be the focus of the forthcoming Sleep-E trial. What is unique to COSI is that the techniques are “packaged” as a resource which is tailored to meet this group of parents' particular needs, based on themes identified via interviews with parents of CWE (Cook et al., 2021). The main areas addressed as part of COSI's development were: (i) the inclusion of specific, desired content; (ii) presenting content in a way that was sensitive to their reported needs; and (iii) personalisation of material.

The specific additional content parents requested was a) help to manage their own and their child's anxieties and b) information about sleep and epilepsy. The latter included addressing the relationship between sleep and seizures and the consideration of SUDEP as well as wanting content to reflect the experiences and “voices” of families of CWE. In addition to the inclusion of pertinent content, conveying families' experiences was also achieved through imaginative, animated videos giving personal perspectives of parents and children, as well as by including trouble-shooting and frequently asked questions (FAQs) pages specifically covering key issues or concerns for this clinical group. As has been found in other studies (e.g., Tan-MacNeill et al., 2020), parents suggested that the inclusion of this relevant content would add credibility to the material and ideas presented as part of COSI and help parents feel that this was something that they could, and wanted to, engage with.

Also important to parents was that there was appropriate presentation of content, which was practical, non-prescriptive and flexible (over time and between families). The authors were mindful of these needs, aiming to empower parents, rather than make them feel as if they had “failed” if they were unable to put a particular strategy in place at a given moment. In adopting this approach, COSI devolves informed management decisions to parents, both about the technique to use and the timing of its use. This can be seen as a step toward increasing levels of parental self-efficacy and/or ownership of decision-making around sleep for parents of CWE, which has been identified as a beneficial approach in paediatric epilepsy care (Berg et al., 2013).

The last main area of development concerned ways to personalise COSI. Primarily this was achieved through embedding functionality to allow the recommendation of specific modules (or advice about when to seek help outside COSI, as appropriate), based on parental responses to a screening questionnaire about their child's sleep. Other more general features of “personalisation” included offering parents choices in how to approach management and specific information about use of COSI in children with co-occurring conditions of Autism, ADHD or intellectual disabilities. Their answers to any quiz questions were also responded to with personalised comments, confirming the correct answer or explaining incorrect answers. The authors were aware that there were additional ways in which COSI could have been personalised (e.g., using the child's name where appropriate) but, at review, this was not considered as necessary by parents; it appeared that “personalisation” of relevant content, rather than in more superficial ways during website/online interaction, was considered important. The importance of customisation of online intervention material has been highlighted in a large meta-analysis where tailored online health behaviour change interventions were found to result in improved health outcomes when compared to control conditions (Lustria et al., 2013). In addition, a customised approach has been shown to be beneficial when delivering sleep interventions in a sample of parents of disabled children (Beresford et al., 2010).

Overall, it is hoped that the measures undertaken in the development of COSI will provide help, information, support and reassurance around sleep to parents of CWE and, perhaps, the general approach to developing a parent resource in co-collaboration with parents, may serve as a useful model for the development of other, condition-specific online BSIs in the future.

While most of the parents' ideas for features of an online BSI were integrated into COSI there were two areas where this was challenging to achieve. For example, some parents suggested having an accompanying, parallel online sleep resource for CWE (Cook et al., 2021). This was extensively considered by the team, however, given the age range of children for which COSI was intended (5–12 years) and the amount of material covered in COSI it was felt that a single “resource for children” would not be practical as multiple, age-appropriate, child COSIs would be required. However, the development, and evaluation, of sleep-related materials designed for CWE is an interesting possibility for consideration in the future. If COSI in its current format, designed for parents of CWE, is efficacious, perhaps developing an accompanying child-specific COSI could be explored to see if this is a helpful addition to improve familial engagement with the materials and overall effectiveness of the intervention.

An additional factor suggested by a small number of parents in the study by Cook et al. (2021) was the inclusion of an interactive component such as a forum or “live” FAQs page. However, while this was considered by the team, ultimately this was avoided as it would preclude any future scaled-up, roll-out of COSI, as an ongoing website moderator would be required. Further, it was difficult to reconcile the evidence-based nature of the advice within COSI, with that which would necessarily be provided by parent anecdote. As such, a decision was taken to restrict this to a non-live FAQ and to include within that some advice for parents about how to engage with material they find on the already available parent forums. However, the inclusion and managing of a “live” or interactive component is one that could be considered in future intervention developments and may further help with parental engagement with the website, particularly as many parents reported that hearing other parents' views and experiences was highly useful and beneficial to them (Wo et al., 2018; Cook et al., 2021).

Parental reviews of the usefulness and functionality of COSI were unanimously positive. We were expectant of an extensive iterative process, involving making multiple rounds of amendments to COSI. However, the first draft version of COSI was viewed by parents as definitely beneficial in terms of its content and usability so that at first review the parental “approval metric” criteria was met. Following this creation and approval of COSI, its effectiveness will be evaluated as part of the upcoming CASTLE Sleep-E trial, where use of COSI will be compared with “standard care” in a randomised controlled-trial. Outcomes including child sleep, cost-effectiveness and a range of child and family functioning measures will be explored (see https://castlestudy.org.uk/). The clinical trial is due to begin participant recruitment of 110 participants in June 2021. It is hoped that COSI will improve the sleep of CWE with sleep problems and, as a result, also contribute to improvements in a range of broad health and well-being outcomes in CWE and their parents. We also hope that COSI will offer busy clinicians, who are aware of the importance of sleep in CWE, a specifically designed resource to which relevant families could be directed for prevention and intervention advice. An online format is scalable, translatable, has potential accessibility benefits for most families, reduces the cost and time of clinical staff's involvement and thus may offer clinical services extra time to focus on the most severe cases or those families who require more clinical input. The recent COVID pandemic emerged just after the CASTLE pilot study had opened and the ability to remotely assign an online sleep intervention was advantageous in these circumstances and will also be used in the upcoming randomised controlled-trial.

A major strength of this study is that the end users of COSI (parents of CWE), and CWE themselves, contributed to the development of COSI and helped to ensure that this online BSI, based on use of evidence-based sleep-behaviour change techniques, included adaptations and additions to meet their needs. A potential limitation of the current study is the small sample size and also that not all parents from phase one (*n* = 9) participated in the evaluation aspect of the trial (*n* = 6). Whilst broad and varied recruitment strategies were employed, recruitment remained a challenge throughout and the final sample was smaller than hoped for. In the current study we intended to use the same participants in phases one and two so that we could be confident that we had interpreted their described needs correctly and that our adaptations to COSI met with their requirements. However, a large sample of different parents will provide feedback on their experiences with using COSI as an intervention in the Sleep-E trial and this will be valuable additional information which could inform future development of COSI more broadly.

Despite the current sample being small it is noteworthy that the parent participants in both phases of the study were mothers of CWE aged 5–15 years and this allowed us to explore their perspectives based on their experiences across childhood. These parents had dealt with a broad range of longstanding sleep-related difficulties and were well-placed to comment about general topics which were relevant for families with CWE. The method of thematic analysis yielded considerable information even from a small sample (Cook et al., 2021). A challenge and further possible limitation of the current study was the need to balance the requirement for evidence-based content with all the suggestions made by parents.

In conclusion, we have outlined the co-creation of COSI, a unique, tailored, online, BSI for use with CWE. We hope that consideration of factors, identified through patient and public involvement, as being salient for parents of CWE will help parent-engagement and optimise COSI's relevance for this patient group. If the results of the clinical trial suggest that COSI is successful in supporting parents to establish healthy sleep in CWE, then the intervention may help to address an unmet need, by offering a low-cost resource to support the many children with epilepsy and their carers who experience poor sleep.

## Data Availability Statement

The datasets presented in this article are not readily available because data are to be made available at the end of the research programme. Requests for access to be made to the Programme Manager: amber.collingwood@kcl.ac.uk.

## Ethics Statement

The studies involving human participants were reviewed and approved by Oxford Brookes University Research Ethics Committee (UREC approval 171108). The patients/participants provided their written informed consent to participate in this study.

## Author Contributions

All authors contributed to conception and design, acquisition of data, analysis and interpretation of data, drafted the article, and revising it critically for important intellectual content.

## Conflict of Interest

The authors declare that the research was conducted in the absence of any commercial or financial relationships that could be construed as a potential conflict of interest.
