# Supplementary material for: Development and Evaluation of the CASTLE Trial Online Sleep Intervention for Parents of Children with Epilepsy
Source: Front Psychol. 2021 Jul 2;12:679804. doi: 10.3389/fpsyg.2021.679804 (PMC8283529; doi:10.3389/fpsyg.2021.679804)
Supplement: Supplementary file 1 [file Data_Sheet_1.pdf]

Supplementary Materials

Appendix 1. Descriptive information about children of the participants

	Age	Gender	Seizure timing	Parent reported sleep problems*
**P1**	10	M	Transitioning between sleep and wake	Current: settling, night waking, sleep-related anxiety, unsettled sleep, co-sleeping<br>Past: sleep terrors, sleep walking
**P2**	15	M	Daytime and during sleep	Current: settling, sleep-related anxiety, night and early morning waking, sleepwalking, sleep terrors, daytime sleepiness<br>Past: room sharing
**P3**	10	F	Transitioning between sleep and wake	Current: night waking, sleep terrors, sleep-related anxiety, co-sleeping<br>Past: morning waking difficulty
**P4**	11	M	Transitioning between sleep and wake	Current: settling, sleep-related anxiety, night waking<br>Past: room sharing
**P5**	5	F	Daytime. 1 year without seizures (due to medication)	Current: settling. night waking, sleep terrors, sleep-related anxiety, room sharing, co-sleeping, daytime sleepiness
**P6**	9	M	During sleep	Current: settling, night and early morning waking<br>Past: daytime sleepiness and co-sleeping
**P7**	13	F	During sleep and transitioning between sleep and wake	Current: settling, morning waking difficulty
**P8**	7	M	Transitioning between sleep and wake	Current: night and early morning waking, co-sleeping

**P9**	13	M	Daytime and transitioning between sleep and wake	Current: settling, night waking, poor sleep quality, daytime sleepiness, possible restless leg syndrome

*Current sleep problems refers to problems which are currently present but it should be noted that the duration of these problems is generally longstanding (since infancy or beyond), with most problems also being also present in the past. Past sleep problems refers to problems which have now resolved but which were significant problems at some point.

Appendix 2. COSI evaluation questionnaire

Section One - The questions in this section ask about how easy or difficult you found it to access and use this online sleep programme.

Item number and question	Response options
**1) Were you able to log on to the intervention website?**	Always<br>Often<br>Sometimes<br>Rarely<br>Never
**2) Were you able to access the webpages of content?**	Always<br>Often<br>Sometimes<br>Rarely<br>Never
**3) Were you able to see the videos?**	Always<br>Often<br>Sometimes<br>Rarely<br>Never
**4) Were you able to hear the videos?**	Always<br>Often<br>Sometimes<br>Rarely<br>Never
**5) Were you able to access the quizzes?**	Always<br>Often<br>Sometimes<br>Rarely<br>Never
**6) Were you able to print off any materials you wanted to?**	Always<br>Often<br>Sometimes<br>Rarely<br>Never
**7) Were you able to find your way/navigate around the sleep programme website?**	Always<br>Often<br>Sometimes<br>Rarely<br>Never
**8) Would you recommend this sleep programme to other families who have children with epilepsy?**	Always<br>Often<br>Sometimes<br>Rarely<br>Never

Section Two - The questions in this section are about the techniques suggested in the sleep programme. In the final version of this sleep programme we will ask parents to tell us, for each technique, if they tried to use it, how helpful they found it and how often they were able to put it into practice. You do not need to tell us these things (since we do not expect you to have tried out the ideas in the sleep programme) but please read the questions and, for each technique we have included, tell us how acceptable you think it is to include this technique in the programme. Even if you personally would not choose to use this technique do you think

it’s OK to suggest that some parents of children with epilepsy might find it helpful for dealing with their child’s sleep difficulties?

For each of the techniques listed below please tell us how acceptable you think it is to include these in the intervention by selecting ONE response for each technique.

Technique	Response options	Follow up
	Do you think the technique is…	If you selected unacceptable or very unacceptable, why do you think this technique is not acceptable?
Make changes to my child’s sleep environment (e.g. curtains, bedding, temperature, noise level)	Very acceptable<br>Acceptable<br>Neither acceptable nor unacceptable<br>Unacceptable<br>Very unacceptable	
Make sure my child’s sleep wake times were regular (e.g. consistent bed and morning wake times)	Very acceptable<br>Acceptable<br>Neither acceptable nor unacceptable<br>Unacceptable<br>Very unacceptable	
Introduce a regular or tweak an existing bedtime routine to include calming activities in lead up to sleep period	Very acceptable<br>Acceptable<br>Neither acceptable nor unacceptable<br>Unacceptable<br>Very unacceptable	
Avoid stimulating things around the sleep period (e.g. computer games or exercise)	Very acceptable<br>Acceptable<br>Neither acceptable nor unacceptable<br>Unacceptable<br>Very unacceptable	
Avoid or reduce child’s intake of food or drinks that contain caffeine	Very acceptable<br>Acceptable<br>Neither acceptable nor unacceptable<br>Unacceptable<br>Very unacceptable	
Remove electronics from your child’s bedroom	Very acceptable<br>Acceptable<br>Neither acceptable nor unacceptable<br>Unacceptable<br>Very unacceptable	
Spend time outdoors and in natural light	Very acceptable<br>Acceptable<br>Neither acceptable nor unacceptable<br>Unacceptable<br>Very unacceptable	
Avoid long or excessive naps	Very acceptable<br>Acceptable<br>Neither acceptable nor unacceptable<br>Unacceptable<br>Very unacceptable	

Limit the amount of time your child is in bed but not sleepy	Very acceptable<br>Acceptable<br>Neither acceptable nor unacceptable<br>Unacceptable<br>Very unacceptable	
Return child to their own bed if they come out of their bed	Very acceptable<br>Acceptable<br>Neither acceptable nor unacceptable<br>Unacceptable<br>Very unacceptable	
Encouraging your child to stay in their room until a set time in the morning	Very acceptable<br>Acceptable<br>Neither acceptable nor unacceptable<br>Unacceptable<br>Very unacceptable	
The camping out method - Staying with my child if they were awake during the sleep period but gradually reducing my presence or contact	Very acceptable<br>Acceptable<br>Neither acceptable nor unacceptable<br>Unacceptable<br>Very unacceptable	
The checking method - Checking my child is they were awake during the sleep period but gradually extending the amount of time between checks	Very acceptable<br>Acceptable<br>Neither acceptable nor unacceptable<br>Unacceptable<br>Very unacceptable	
The bedtime pass method - Giving my child a ‘bedtime pass’ which they can use to come out of their room once or twice during the settling period	Very acceptable<br>Acceptable<br>Neither acceptable nor unacceptable<br>Unacceptable<br>Very unacceptable	
The bedtime fading method - Setting the time my child is naturally falling asleep as their temporary bedtime and then moving the bedtime earlier once they are happily settling	Very acceptable<br>Acceptable<br>Neither acceptable nor unacceptable<br>Unacceptable<br>Very unacceptable	
Rewarding my child to encourage positive sleep behaviour	Very acceptable<br>Acceptable<br>Neither acceptable nor unacceptable<br>Unacceptable<br>Very unacceptable	
Limit setting or ignoring child’s demands if it is safe to do so e.g. requests for another drink or story	Very acceptable<br>Acceptable<br>Neither acceptable nor unacceptable<br>Unacceptable<br>Very unacceptable	
Get your child to draw or write down any worries they may have	Very acceptable<br>Acceptable<br>Neither acceptable nor unacceptable<br>Unacceptable<br>Very unacceptable	
Using relaxation/visual imagery techniques	Very acceptable<br>Acceptable<br>Neither acceptable nor unacceptable	

	Unacceptable<br>Very unacceptable	
Using social stories, role play or imagination techniques	Very acceptable<br>Acceptable<br>Neither acceptable nor unacceptable<br>Unacceptable<br>Very unacceptable	
Anything else? If so, please describe…	Very acceptable<br>Acceptable<br>Neither acceptable nor unacceptable<br>Unacceptable<br>Very unacceptable	

SECTION THREE: We hope to include questions (similar to the questions in Sections One and Two) in our final online sleep programme so that we can know if parents are finding the sleep programme helpful and easy to use. Please answer the questions below, in Section three, to tell us what you think about the questions we hope to ask.

**Item number and question**	**Response options**
1) Do you think that the time it takes to complete the questions is	Far too long<br>A little too long<br>About right<br>A little too short<br>Far too short
2) We want parents to be able to understand the questions we ask. How do you find the wording/language used in the questions?	Very difficult [please specify any specific words or language]<br>Difficult<br>About right<br>Easy<br>Very easy
3) Do you think there are any other questions we should be asking too, so that we can better understand parents' experience of using this sleep programme?	Yes [If so, please provide any relevant examples]<br>No
4) If there is anything else you would like to tell us or any other comments that you wish to add please include them here:	

Thank you very much for taking the time to complete these questions about the Castle Online Sleep Intervention. Your participation and feedback is incredibly useful.

If you have any further questions or wish to provide any additional feedback please do not hesitate to contact Luci Wiggs (lwiggs@brookes.ac.uk).

Appendix 3. Child sleep interview schedule

How do you feel about going to bed/sleep?

What sleep problems, if any, would you most like help with?

Do you have any problems with going to bed/sleep?

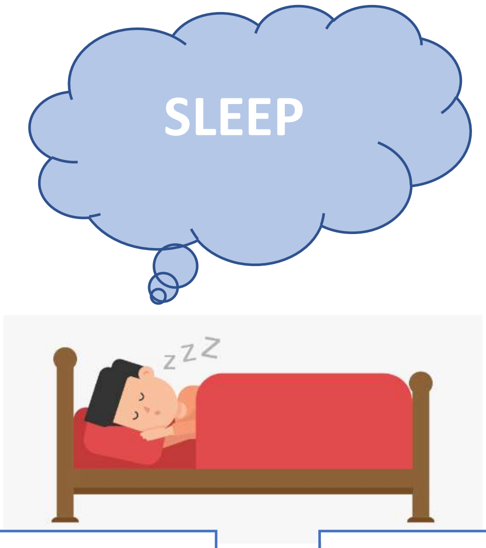

Are there any special problems because of your epilepsy/seizures?

Are there any things you've tried to help you with sleep? What things were helpful? Why?

What things were not helpful? Why?

Is there anything else about your sleep that you think is important but hasn't been mentioned?
